# Walk this way: modeling foraging ant dynamics in multiple food source environments

**DOI:** 10.1007/s00285-024-02136-2

**Published:** 2024-09-12

**Authors:** Sean Hartman, Shawn D. Ryan, Bhargav R. Karamched

**Affiliations:** 1 College of Music, Florida State University, Tallahassee, FL 32306 USA; 2 Department of Mathematics and Statistics, Cleveland State University, Cleveland, OH 44115 USA; 3 Center for Applied Data Analysis and Modeling, Cleveland State University, Cleveland, 44115 OH USA; 4 Department of Mathematics, Florida State University, Tallahassee, FL 32306 USA; 5 Institute of Molecular Biophysics, Florida State University, Tallahassee, FL 32306 USA; 6 Program in Neuroscience, Florida State University, Tallahassee, FL 32306 USA

**Keywords:** Self-organized systems, Collective behavior, Trail formation, Lattice model, Dispersion relation, 92-10, 92D40, 35P05, 60G99, 82M60

## Abstract

Foraging for resources is an essential process for the daily life of an ant colony. What makes this process so fascinating is the self-organization of ants into trails using chemical pheromone in the absence of direct communication. Here we present a stochastic lattice model that captures essential features of foraging ant dynamics inspired by recent agent-based models while forgoing more detailed interactions that may not be essential to trail formation. Nevertheless, our model’s results coincide with those presented in more sophisticated theoretical models and experiments. Furthermore, it captures the phenomenon of multiple trail formation in environments with multiple food sources. This latter phenomenon is not described well by other more detailed models. We complement the stochastic lattice model by describing a macroscopic PDE which captures the basic structure of lattice model. The PDE provides a continuum framework for the first-principle interactions described in the stochastic lattice model and is amenable to analysis. Linear stability analysis of this PDE facilitates a computational study of the impact various parameters impart on trail formation. We also highlight universal features of the modeling framework that may allow this simple formation to be used to study complex systems beyond ants.

## Introduction

Social insect behavior has fascinated the biological community for a long time. The collective behavior of insects can be used to achieve more complicated outcomes than possible for individuals. This is especially important for ant species that rely on communication via pheromone to coordinate activity. In the absence of an external motivation or central control center, ants can perform complex sets of tasks (Dorigo and Birattari 2010; Doerr et al. 2012) and exhibit macroscopic emergent behavior (Couzin and Franks 2003; Deneubourg and Goss 1989). This makes them an ideal model organism for studying the physical origins of self-organizing behavior. For example, in order to find food for survival, ant colonies send foragers away from the nest executing a random search process (Ryan 2016). Once food is found the ants secrete a pheromone to attract other nearby foragers which eventually leads to the formation of a trail (e.g., Ryan (2016); Sumpter and Beekman (2003); Dussutour et al. (2009); Couzin and Franks (2003); Franks (1985); Garnier et al. (2009, 2013); Perna et al. (2012) among others). The ants proceed to break down the food source until interruption (i.e., by a predator or weather) or completion before returning to the colony.

The trail formation is a rich source of collective behavior observations as ants self-organize into lanes along the trail for optimal resource transport. While fascinating to watch, there is still much to be understood about the raiding process—specifically, the raiding process in the presence of multiple food sources. What makes this challenging to model is that individuals within an ant colony cannot directly communicate and instead rely on chemical signaling through pheromones. Thus, in the presence of multiple signals how will the colony proceed through the raiding process to ensure an efficient acquisition of resources?

A given colony of ants provides an ideal biological system to study self-organization because all participating agents are identical and have the same motivations: discover food and break it down for retrieval (Bonabeau et al. 1997). The results derived here provide foundational insight into similar social insect systems such as locusts (Dyson et al. 2015; Ariel et al. 2014) and termites (Calenbuhr and Deneubourg 1992). Though detailed observations of ant foraging behavior have become increasingly available over the last century (cf Schneirla (1940a)), mathematical models are still striving to uncover the underlying behavior of this natural phenomenon. The desire to develop a mathematical model for the foraging/raiding process is motivated by the lack of reproducibility in experiments and the inability to isolate individual factors in the ant dynamics that may reveal the true underlying dynamics. Previous mathematical models have implemented many varying approaches to investigate the dynamics of the ant raiding process. Past approaches include continuum PDEs (Amorim 2015; Johnson and Rossi 2006; Watmough and Edelstein-Keshet 1995a, b; Schweitzer et al. 1997), agent-based models through systems of coupled ODEs (Couzin and Franks 2003; Ryan 2016; Baumgartner and Ryan 2020; Boissard et al. 2013; Vittori et al. 2006; Malíčková et al. 2015), and lattice models where ants move on a discrete grid (Deneubourg et al. 1989, 1990; Solé et al. 2000).

Continuum PDE models forming a system for the ant density and chemical pheromone have shown great qualitative agreement with foraging behavior in ants and allow for rigorous mathematical analysis (Amorim 2015; Alonso et al. 2016; Ramakrishnan et al. 2014), but the microscopic details of those interactions are lost in the process. Individual-based models have successfully captured individual ant movement by prescribing how each member of the colony behave—even going so far as to separate ants into foragers and returners giving each similar dynamics but different motivation for movement (Ryan 2016). Furthermore, these models allow the ants themselves to lay the directed pheromone trail to attract others to a given location (Boissard et al. 2013; Ryan 2016). These individual-based models also show local traffic dynamics analogous to pedestrians at a crosswalk leading to optimized flow of traffic and resources (Couzin and Franks 2003) as seen experimentally (Dussutour et al. 2009). Prior lattice models such as Denuebourg et al. were pioneering for trail formation by using a probabilistic framework to derive multiple trails where ants would pick their next direction based on a biased probability distribution (Deneubourg et al. 1989). This was guided by experimental observation and helped explain diverging patterns at two branches of a land bridge where ants communicate only via pheromone (Deneubourg et al. 1990). Lattice models have also been used to show that ants highly optimize their trail and dynamics while raiding (Solé et al. 2000). Each of these mathematical approaches has revealed interesting features of the raiding process, but none has been dedicated to simultaneous trail formation at multiple food sites.

The primary focus of this work is to develop deeper understanding of ant foraging dynamics by developing a model that incorporates the best features of the lattice-based and agent-based prior approaches. While building upon recent work, the model here is designed to study trail formation in the presence of multiple food sources, which present many interesting questions with biological and mathematical implications. We then rigorously investigate the model through numerical means and reveal underlying properties through the analysis of the corresponding macroscopic PDE formulation. For example, in the presence of a colony’s discovery of multiple food sources, how does a colony allocate its members to retrieve food in an efficient way? One challenge about studying this problem experimentally is that observations suggest that different food sources elicit different recruitment responses indicating that the ants rely on their ability to detect pheromone gradients (Hölldobler 1976).

We emphasize that our model is intended to be as universal as possible whilst remaining realistic. The primary mechanism of collective behavior in our model is chemotaxis. This is not specific to ants, as it can just as easily be applied to study bacteria (Berg 1975; Lux and Shi 2004; Celani and Vergassola 2010) and slime molds (Cohen and Robertson 1971; Ueda et al. 1975; Boussard et al. 2021), amongst many other organisms. However, we apply facets of chemotaxis to unveil a relatively simple manner by which emergent spatiotemporal ordering (trail formation) may occur in ants. Our lattice model framework is thus easily amenable to study other organisms of interest as well.

In Sect. 2, we introduce a novel lattice-based model for ant movement in a domain. The primary new feature is the incorporation of principles from agent-based models for ants (e.g., Ryan (2016); Baumgartner and Ryan (2020)) such as direct pheromone deposition by the returning ants at exponentially decreasing quantities as an individual moves away from the food source. In addition, the pheromone concentration is governed by a reaction-diffusion PDE first introduced in Ryan (2016). The key assumption is that foraging and returning ants are motivated by different factors and therefore are guided by different rules for their dynamics. Once the lattice model is established, we describe a complementary macroscopic PDE system governing ant density analogous to past approaches (e.g., Boissard et al. (2013)). In Sect. 2.1, we provide details of the computational setup of the numerical simulations.^1^ for the lattice model and the macroscopic PDE model. In Sect. 3.1, we perform linear stability analysis on the macroscopic PDE model. We establish a key result that a uniform spread of ants in a domain with a food source is unstable for a large region of parameter space and, furthermore, show numerically that the stable observable is in fact trail formation. More precisely, we demonstrate numerically that the presence of food is a *necessary* but not a *sufficient* condition for destabilization of a homogeneous stationary state. The model is then used to derive enhanced understanding of the multiple food source case which has not been studied extensively in the literature. Understanding this scenario is essential to forming more complete knowledge of ant raiding behavior and provide insight into collective dynamics of social insects as a whole. We conclude in Sect. 4, where we highlight new biological understanding derived from modeling and simulation predictions. We also setup potential future avenues of investigation.

## Bio-inspired lattice model

There are challenges related to modeling ant foraging dynamics and trail formation. Namely, the trail formation can depend on model parameters such as distance from the nest, density of the food source and the size of the food source (Hölldobler 1976). We forgo the latter of these issues by assuming that each food source contains an infinite reservoir of food. In the following we describe a simple lattice model capturing essential features of ant foraging dynamics whilst forgoing more realistic microscopic details. We find that our simple lattice model produces results akin to what is seen in more complicated agent-based models (Ryan 2016). Our model is also amenable to analysis at cost of some fidelity to reality.

### Lattice model description

We model the general terrain as an $M \times N \subset \mathbb {N}^2$ lattice and the ants as $n\in \mathbb {N}$ particles hopping along the lattice nodes. We assume that the timescale of trail formation is small enough (approximately 4–8 h from biological observation of army ants *E. burchellii* (Schneirla 1940b, 1971)) that we ignore births and death in the colony. There is no direct communication between individuals, but rather a response to a chemical pheromone gradient if present represents the only means of (indirect) communication. We designate a single site as the nest, $\textbf{x}_0$. Initially, all *n* ants occupy that designated site. To understand how the location of food sources relative to the ant nest affects spatiotemporal structure of ant motion, we also randomly designate $\mathcal {N} \in \mathbb {N}$ sites as food sources. We assume the colony of ants are particles represented by a set of points $\{\textbf{x}_i\}$, $i = 1,..., n$, where $\textbf{x}_i \in [1,M] \times [1,N]$. Each point can be thought of as the location of the center of mass for an individual ant. We assume the boundaries are reflecting for ants to maintain a fixed population^2^.

**Fig. 1:**
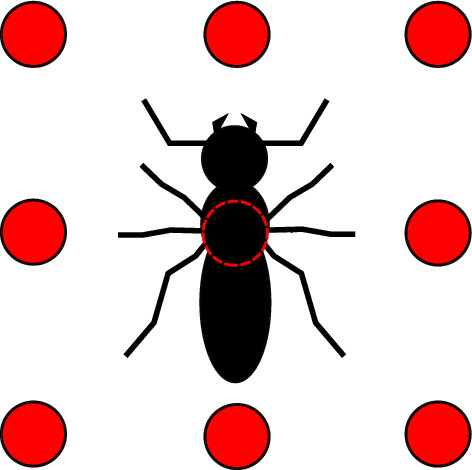
Illustration of an ant on the lattice. We omit the details of an individual ant’s body such as its three components and individual appendages/antennae in the model. Rather we simulate the change in a given ants center of mass across the lattice network. This remains faithful to the biology by considering many ants in a large computational domain where the individual microscopic details becomes less relevant to understanding the macroscopic behavior

Ant motion is subdivided between two types of ants: (i) foraging ants and (ii) carrying ants. Foraging ants are those that are searching for a food source. They undergo an unbiased random walk (Popp and Dornhaus 2023; Charikar et al. 2011). That is, a foraging ant at a given site moves to any adjacent site in its Moore neighborhood with equal probability (Fig. 1). In particular, the angular deviation between fixed time steps was measured experimentally in Pharaoh ants and shown to be well approximated by a normally distributed random variable around the nest (Bicak 2011). At each time step an ant picks a new site to move to based on this probability and thus all ants move with a constant speed. A foraging ant becomes a carrying ant once it locates a food source. Its dynamics are no longer stochastic. Upon reaching the nest, a carrying ant again becomes a foraging ant.

Recent works have incorporated two pheromones into modeling frameworks: one where ants carrying food mark a food source (Dussutour et al. 2009; David Morgan 2009) and another where foraging ants mark the location of the nest (Steck 2012; Jackson et al. 2007; Ramirez et al. 2018). We assume throughout this work that carrying ants know the location of their nest and we choose to forgo the second pheromone in the model. Thus, we only implement the net result of the second chemical deposition by assuming that individual returning ants take the direct path home toward the nest as has been observed experimentally in Wehner (2003); Müller and Wehner (1988); Buehlmann et al. (2014); Narendra et al. (2013). Here it was observed experimentally that even obstructing ants by imposing barriers after a food source is located does not dissuade them from following the most direct path home. This suggests the pheromone alone may aid in finding the nest, but a more complex neurological process may contribute as well. In these works, it is noted that ants can follow landmark routes and recognize locations to navigate. The evidence suggests that ants use path integration and their collective knowledge of complex outbound routes to triangulate the terrain and return home directly. Ants do not use complicated path integration akin to humans, but rather use an approximation accounting for navigational errors (Müller and Wehner 1988).

To recruit additional ants to the food source a given ant found, a carrying ant secretes pheromone in exponentially decreasing amounts as it travels to its nest to form a chemical trail from the nest to the food source. Though decreasing we assume ants keep secreting pheromone until they reach the nest in contrast to another recent work that has considered ants having a finite chemical supply until they “recharge" at the nest site (Malíčková et al. 2015). The physical response of an ant to a chemical stimulus is well documented (Calenbuhr and Deneubourg 1992; Couzin and Franks 2003; Panait and Luke 2004). Following Weber’s Law, foraging ants can then use their antennae to analyze the local concentration of pheromone and preferentially travel against the concentration gradient to reach a food source more efficiently. Recent models assume that to impose Weber’s law the ant’s response rate only depends on the pheromone concentration at the tips of the antennae referred to as *tropotaxis*. The rate of pheromone secretion is assumed to diffuse and decrease exponentially with a carrying ant’s distance from the food location (Couzin and Franks 2003; Robinson et al. 2008). Pheromone deposition and trail laying are modeled by a two-dimensional reaction-diffusion process for the chemical concentration $c(\textbf{x},t)$ first introduced in Ryan (2016):

1 $$\begin{aligned} \frac{\partial c}{\partial t} - {{D}{\Delta _{ij}} c} + \gamma c = \sum _{k=1}^{K}\sum _{j=1}^{J} {Ae^{-\Big ({\frac{||\textbf{x}_j(t) - \textbf{x}_f^{(k)}||}{\sigma }}\Big )^2}}{\delta {(\textbf{x} - \textbf{x}_j(t))}}. \end{aligned}$$

Here $\textbf{x}_f^{(k)}$ is the location of the *K* food source(s), *J* is the number of carrying ants, $\Delta _{ij}$ is the discrete Laplacian, *D* is the diffusion coefficient controlling the rate at which the pheromone spreads, and $\gamma$ is the evaporation coefficient that ensures an exponential decay of the pheromone in time. The coefficient $Ae^{-\Big ({\frac{||\textbf{x}_j(t) - \textbf{x}_f^{(k)}||}{\sigma }}\Big )^2}$ represents the amount of pheromone deposited at time *t* and decays as a carrying ant moves away from the food source. This decrease is needed to ensure that the proper gradient forms due to the competition with diffusion. The parameter $\sigma$ characterizes the strength of the decay in pheromone production as ants travel away from the food source. In the following, we set $\sigma = 1$ so that decay length coincides with lattice spacing.

**Table 1:** Values used in simulation for dimensionless biological parameters

Parameter	Non-dim value (Sim)	Physical description	Biological value
*L*	1.0	Effective ant length/lattice spacing	1 cm
*dt*	0.001	Time step	1 s (Ryan 2016; Amorim 2015)
*N*	1000	Amount of ants in a given colony	$10^2$–$10^6$ (supercolonies (Baumgartner and Ryan 2020))
*D*	10.0	Pheromone diffusion coefficient	0.01 cm (Couzin and Franks 2003; Calenbuhr and Deneubourg 1992)
$\gamma$	0.001	Pheromone degradation coefficient	1/300 s (Ryan 2016; Couzin and Franks 2003)
*A*	1.0	Amount of pheromone deposited	g cm (Couzin and Franks 2003; Calenbuhr and Deneubourg 1992)
$\varepsilon$		Pheromone conc. detection threshold	Estimated

The initial distribution of chemical is taken as zero so there is no pre-defined directional preference. We prescribe homogeneous Fourier-type boundary conditions for the pheromone so that we have

$$\begin{aligned} -D\frac{\partial c}{\partial x} \Big |_{x=0}&= - c(0,y,t)\\ -D\frac{\partial c}{\partial x} \Big |_{x=M}&= c(M,y,t)\\ -D\frac{\partial c}{\partial y} \Big |_{y=0}&= - c(x,0,t)\\ -D\frac{\partial c}{\partial y} \Big |_{y=N}&= c(x,N,t). \end{aligned}$$

Hence, Fickian flux along the boundary is preserved. That is, along the boundary, the chemical continues to flow with its concentration gradient.

### Simulation

On each time step, one of the ants is randomly selected to move to one of its neighboring sites according to rules described below. We define *n* such ant movements as one unit of time. Initially, all ants are located at the nest, $\textbf{x}_0$. At all points in time, we track foraging ants and carrying ants at each location. One can find the simulation parameters and the corresponding biological values taken from experimental observation in Table 1. We describe the simulation in detail in the following.

**Foraging Stage.** During this stage all ants undergo a biased random walk. More specifically, when an ant is selected to move, and no other ant has found food, it moves into any one of the adjacent sites in its Moore neighborhood with equal probability (Amorim 2015; Boissard et al. 2013; Schweitzer et al. 1997). This continues until one of the ants finds a food source. Thereafter, pheromone detection biases the random walk that foragers undergo (see below).

**Pheromone Secretion** Once an ant finds a food source, $\textbf{x}_f$, it immediately starts secreting pheromone which attracts other ants (Jackson and Ratnieks 2006; Steck 2012). The dynamics of the pheromone concentration field, $c(\textbf{x},t)$, are simulated by Eq. (1). We solve Eq. (1) with a Forward Euler discretization and take the numerical time step $\Delta t = 0.001$ units and spatial step $\Delta x = 1$ unit.

**Trail Formation Stage.** We assume that ants that reach a food source begin carrying food. Such carrying ants make a beeline (direct path) for the nest (Müller and Wehner 1988; Wehner 2003; Wehner et al. 2016). That is, when a carrying ant is selected to move during a simulation, it moves to the first adjacent site encountered when extending the vector $\textbf{x}_0 - \textbf{x}_f$ from the center of the site where the carrying ant is selected. The actual movement of a carrying ant will not resemble a beeline on the lattice, but rather an approximation and therefore the lattice model accounts for short length scale randomness in the returning trajectory. However, as lattice size (number of lattice points) is increased, this method will result in a more direct path trajectory for carrying ants returning to the nest.

Foraging ants subject to the pheromone concentration field now undergo a biased random walk. The probability that a foraging ant moves to an adjacent site in its Moore neighborhood is weighted according to the concentration of pheromone at that site relative to the pheromone concentration at the current location of the ant. This is consistent with recent studies showing indirect decision making by individual ants in response to local pheromone gradients (seen both in experiments of Couzin and Franks (2003); Mokhtari et al. (2022) and theoretical studies (Panait and Luke 2004)). To implement this, we compute the difference in pheromone concentration at the current location of the ant, $\textbf{x}_c$, and the pheromone concentration at a given adjacent site in the Moore neighborhood of the ant, $\textbf{x}_a$. Here, $a \in A$ and $c \notin A$, where *A* is the set of locations in the Moore neighborhood of the ant selected to move. Thus, $\textbf{x}_c \ne \textbf{x}_a$. Let $\Delta c_a \equiv c(\textbf{x}_a,t) - c(\textbf{x}_c,t)$. We assign a weight, $\mathcal {W}_a$, to each site as follows:

$$\begin{aligned} \mathcal {W}_a = \left\{ \begin{array}{cc} \varepsilon , & \text {if} \quad \Delta c_a < 0\\ 1, & \text {if} \quad \Delta c_a = 0\\ 1 + \Delta c_a, & \text {if} \quad \Delta c_a > 0 \end{array} \right. \end{aligned}$$

The probability that a foraging ant subject to a pheromone concentration field moves from $\textbf{x}_c$ to $\textbf{x}_a$ is then

$$\begin{aligned} \mathcal {P}_{\textbf{x}_c \rightarrow \textbf{x}_a} = \frac{\mathcal {W}_a}{\sum _{k \in A} \mathcal {W}_k}. \end{aligned}$$

Thus, a foraging ant subject to a pheromone concentration field preferentially moves against the largest pheromone concentration gradient, whilst still having a nonzero probability of moving in neutral directions. We assign a small weight $\varepsilon$, with $0<\varepsilon \ll 1$, describing the probability of an ant to move down the concentration gradient of a pheromone. The $\varepsilon$ parameter ensures an ant will not get stuck in a local maximum concentration at a lattice point. In this case, every direction would be assigned $\varepsilon$ and therefore it would pick a random neighboring lattice point to move to. This may occur due to the random fluctuations in the motion of the carrying ants as they deposit pheromone coupled with the diffusion into the surrounding environment. This has no effect on the global behavior and is added to make numerical simulations of the lattice model more robust. We note that this situation occurred rarely in simulation where the chemical magnitude was extremely small and had no effect on the global macroscopic behavior of the colony. This is consistent with a recent observation in Wyatt et al. (2003) where they found that there is a minimum concentration threshold below which ants cannot sense and therefore would return to a random walk motion.

*Remark.* We note here that numerical solution of Eq. (1) occurs concurrently with updating ant positions. Because ant position updates occur on a unit time scale and Eq. (1) updates occur on a time scale prescribed by the time step $\Delta t$, care must be taken in implementing the simulation. Once Eq. (1) becomes active at the arrival of an ant to a food source, we first update the PDE and then update ant positions.

We also note that the results presented for the stochastic model herein correspond to large population size systems. Trail formation in small population stochastic systems can be more challenging since pheromone secretion can fluctuate. However, in the large population size regime, pheromone secretion contracts to the mean, and the formation of trails is better seen and understood.

### Lattice model results

More realistic models of foraging ant dynamics show that following a transient phase wherein the distribution of ant positions is approximately Gaussian, ants form a robust trail from the nest to a located food source (Ryan 2016; Sumpter and Beekman 2003; Dussutour et al. 2009; Couzin and Franks 2003; Franks 1985; Garnier et al. 2009, 2013; Perna et al. 2012). In the following, we validate our model against the one presented in Ryan (2016) and then show that our model using parameters in Table 1 can produce more sophisticated results that capture foraging ant behavior seen in experiment or nature. The beauty of our simple model is that it coarse-grains over microscopic underpinnings described in other models to capture the macroscopic picture but nevertheless captures details described in more specific models (see (Amorim et al. 2019; Malíčková et al. 2015; Fontelos and Friedman 2015; Vela-Pérez et al. 2015), for example). We validate against (Ryan 2016) due to the direct relation between our model and the one presented there. To consider the lattice model (or any model of ant dynamics and trail formation) a success it must at minimum capture three distinct regimes: (i) the colony forages within an area of a given size around a central nest, (ii) the model has the ability to recruit ants to any food sources discovered, and (iii) the model exhibits complex macroscale patterns of trails emanating from the nest (Ramirez et al. 2018; Wyatt et al. 2003).

**Validation.** To validate our model, we compare our results with those presented in a more realistic agent-based model (Ryan 2016; Baumgartner and Ryan 2020) and experiment (e.g., Couzin and Franks (2003)). In Ryan (2016), Ryan presents a detailed agent-based model describing ant foraging dynamics. There, before any food is found, the distribution of ants about the nest is approximately Gaussian. Once a food source is found, pheromone secretion ensues and there is a rapid transition to a state where an ant trail connecting the nest to the food location forms. Ryan also showed that the interior of well-formed trails consisted of returning ants and the exterior of foraging ants.

**Fig. 2:**
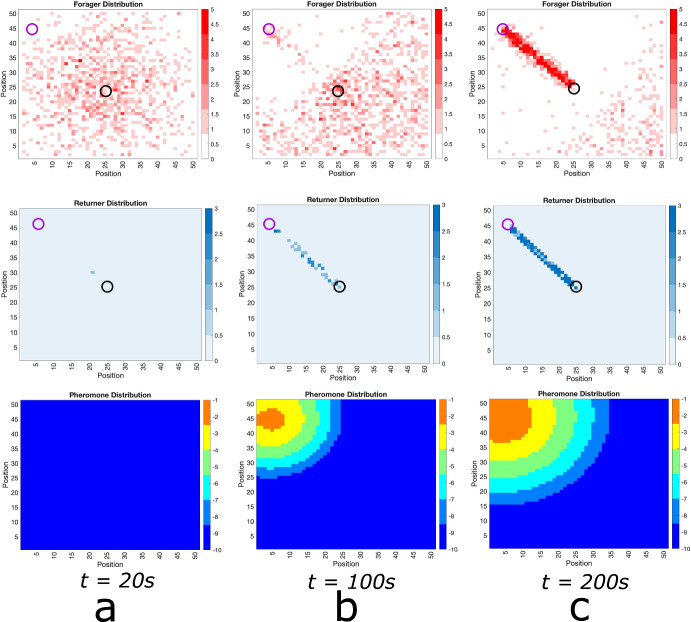
Foraging Ant Dynamics: One Food Source. Top row: foraging ant distribution. Middle row: returning any distribution. Bottom row: pheromone distribution (plotted in log scale). **a** The foraging ants distribution is approximately Gaussian around the nest (center black circle) until food source (purple circle) is discovered, whence pheromone is released as returners move toward the nest. **b** The pheromone excretion initiates emergent self-organization of the ants into a trail connecting the nest to the food source. **c** The trail is well-developed. Due to the fact that the food at the source does not diminish, this trail continues forever (color figure online)

In Fig. 2, we show sample simulations of our lattice model that depict the same behavior. Initially, the distribution of foraging ants about the ant nest is approximately Gaussian, stemming from their dynamics being a discrete random walk (Fig. 2a. Once a food source is located and pheromone secretion is underway, ants begin to self-organize and form a trail connecting the nest to the food source (Fig. 2b. Eventually, the vast majority of the ants partake in a well-formed trail between the nest and the food source (Fig. 2c).

**Fig. 3:**
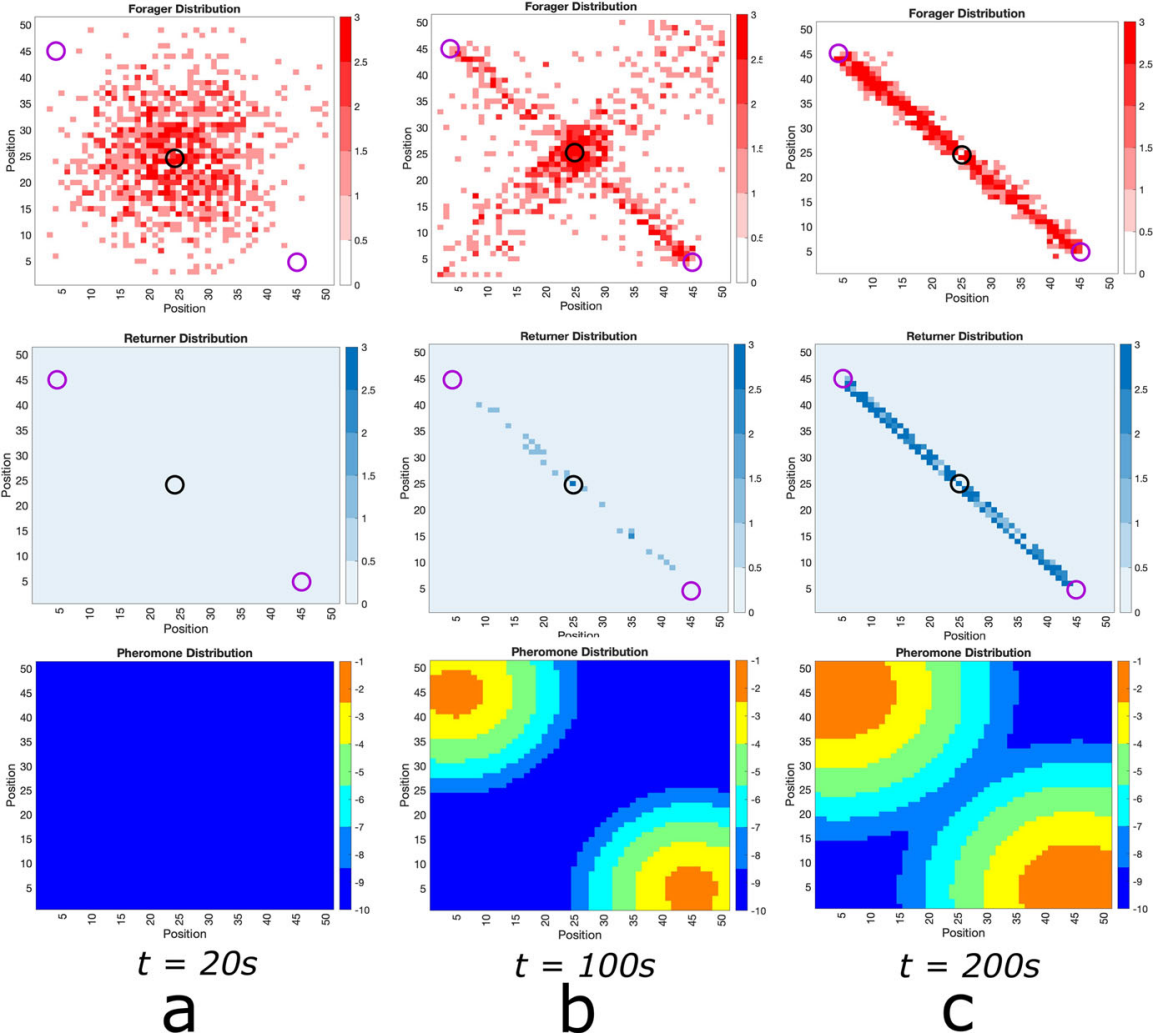
Foraging Ant Dynamics: Two Equidistant Food Sources. Top row: foraging ant distribution. Middle row: returning ant distribution. Bottom row: pheromone distribution (plotted in log scale). **a** The foraging ants distribution is approximately Gaussian around the nest (center black circle) until the food sources (purple circles) are discovered, whence pheromone is released as returners move toward the nest. **b** The pheromone excretion initiates emergent self-organization of the ants into trails connecting the nest to the food sources. **c** The trails are well-developed. The food source does not diminish and there is no asymmetry in the properties of the food sources relative to the ant nest, both trails continue forever (color figure online)

Furthermore, returning ants in a well-formed trail follow a direct path (Fig. 2c, middle row), whereas foraging ant locations (top row) are distributed around the main trail. Hence, returning ants are definitively found in the interior of well-formed trails whereas foraging ants can be found on the exterior. Thus, our simple lattice model reproduces the results of the more realistic agent-based model of Ryan. We also note here that the results are consistent with experimental results (Couzin and Franks 2003; Perna et al. 2012). In particular, the foraging trail is wider resulting in more ants on the outside of the central trail lane. This exactly matches the qualitative observations of ants in the experimental observations of Couzin et al. Couzin and Franks (2003) and it is remarkably captured in our simple lattice model Fig. 2c.

**Multiple Food Sources.** The detailed agent-based model presented in Ryan (2016) was lacking in one regard: when multiple food sources were present, the model did not allow for the persistence of multiple ant trails connecting the nest to each food source. Experimental studies, however, clearly show that ant colonies establish multiple ant trails in the presence of multiple distinct food sources (Burns et al. 2021).

Our simple lattice model allows for the persistence of multiple ant trails in multiple food source environments. In Fig. 3, we show snapshots of foraging ant dynamics in an environment consisting of two equidistant food sources from the ant nest. Initially, there are no returning ants and the foraging ant distribution is approximately Gaussian (Fig. 3a. Once the food sources are found, the ants begin to self-organize into two distinct raiding pathways (Fig. 3b and then establish well-formed trails connecting the nest to the food sources (Fig. 3c.)

**Fig. 4:**
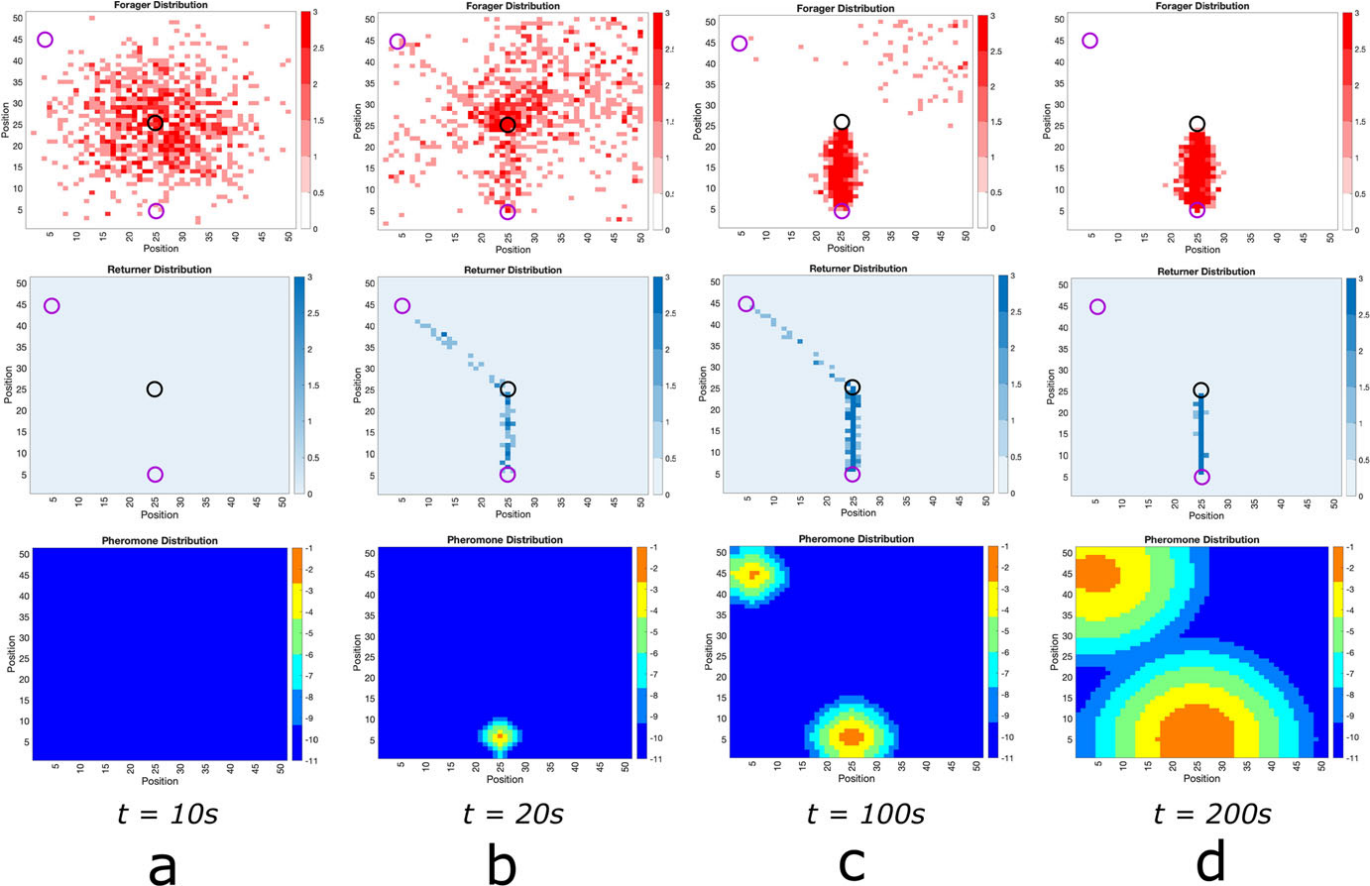
Foraging Ant Dynamics: Two Food Sources: One closer, one far. Top row: foraging ant distribution. Middle row: returning any distribution. Bottom row: pheromone distribution (plotted in log scale). **a** The foraging ants distribution is approximately Gaussian around the nest (center black circle) until the food sources (purple circles) are discovered, whence pheromone is released as returners move toward the nest. **b** The pheromone excretion initiates emergent self-organization of the ants into trails connecting the nest to the food sources. **c**, **d** Both trails are well-developed at short time scales, but because it is less efficient to send ants to the farther food source and less ants visiting leads to the pheromones on that trail dissipating faster. Therefore, eventually all ants converge to the trail connecting the nest to the closer food source. Because food at the source does not diminish, this trail continues forever (color figure online)

If two food sources are present but not equidistant from the ant nest, the results are similar (see Fig. 4). The main difference is that the state in which multiple trails connecting the nest to the distinct food sources manifests as a *quasistationary* state (Figure 4c before all ants converge to the trail connecting the nest to the nearer food source (Fig. 4d. In this context, the disparity in distances to distinct food sources renders the use of ants to obtain food from farther away inefficient. In this situation, the model predicts that ants will preferentially collect all the food from the nearer food source before traveling farther for food. Even in the case presented in Fig. 4, wherein the farther food source is just one unit farther than the closer food source, the asymmetry in the system is sufficient for the ant colony to focus all of its population on the closer food source. This can be explained simply by appealing to diffusion correlation length of the pheromone signal. This length is defined by the distance from the trails the pheromone can effectively be felt by a foraging ant^3^. The closer food source manifested a stronger pheromone signal near the nest, which in turn resulted in stronger ant recruitment. The recruitment then facilitated a stronger pheromone gradient signal connecting the nest to the closer food source (positive feedback). Both aspects of this process each reinforce themselves.

This latter effect is observed in “winner-take-all" processes in biology, wherein identical targets compete for a limited resource of nutrients. The emergence of some asymmetry in the system eventually pushes all the resources to be held by one of the targets. One example of such a process is neurite polarization, wherein one of several identical protrusions (neurites) emanating from a nascent neuron compete for a common resource (such as tubulin). Due to intracellular and thermodynamic noise, one protrusion collects more resource than others by chance. This single neurite becomes the axon of the mature neuron, while the others become dendrites (Bai et al. 2023; Toriyama et al. 2010; Schelski and Bradke 2017).

Such processes are also observed in natural and synthetic gene circuits that involve mutual inhibition positive feedback loops (Alon 2019; Bertram 2015; Sadeghpour et al. 2017). A motif of such systems is bistability—wherein at equilibrium one protein persists and the other goes extinct. The persistence of either protein in the system is equally likely, but initial conditions drive the system one way or the other. Such systems are called “toggles” (Tyson et al. 2003), as varying key biophysical parameters can shift the equilibrium to a state where the other protein persists. Winner-take-all mechanisms are also present in learning in neural networks (Kaski and Kohonen 1994; Maass 2000) and synthetic gene circuits (Stone et al. 2022), among many other systems.

In this perspective, ants can be construed as resources and the food sources as targets. When there is nothing differentiating the identical food sources (as in Fig. 3), ants form longstanding trails to both food sources. When asymmetry in the distances to the respective food sources is present, however, the closer food source eventually collects all the ants.

Thus, our simple lattice model consisting of only essential basic features of foraging ant dynamics produces results that are observed in more realistic agent-based models and in experimental studies. One feature of the lattice model is that it is simple to implement. Another feature is that it is amenable to analysis, which we discuss in the next section.

## Macroscopic probabilistic model

To understand the dynamics of the stochastic lattice model we develop a macroscopic equation describing the evolution of probability densities of foraging and carrying ants. Although the simulated system is spatially discrete, the corresponding discrete master equation is cumbersome and unintuitive. In the limit of a very large lattice size and small grid size, the master equation is well-approximated by the corresponding macroscopic PDE. The PDE presented here is not a systematic derivation from a master equation description of the transitions between the microscopic configurations of the lattice. Rather, we present a PDE that captures basic features of the stochastic lattice model. We first verify that the qualitative behavior of the PDE coincides with that of the stochastic lattice model before performing analysis on it to divulge the impact various biophysical parameters play in the stability of stationary states. The systematic derivation of an exact continuum limit of this stochastic lattice model and the precise mappings between model parameters in the stochastic lattice model and the continuum limit remain an open question.

We present the model for a single food source, but it is easily generalized to multiple food sources. Let $p(\textbf{x},t)$ denote the probability density for the position of a foraging ant at time *t* and let $q(\textbf{x},t)$ denote the probability density for the position of a carrying ant at time *t*. Finally, let $u(\textbf{x},t)$ be the continuum analog of $c(\textbf{x},t)$. Then, the dynamics *p*, *q*,  and *u* can be characterized by

2 $$\begin{aligned} \frac{\partial p}{\partial t}&= \alpha \Delta p - \sigma \nabla \cdot ((\nabla _{\textbf{x}} u) p) - \Omega _{p\rightarrow q}\delta (\textbf{x} - \textbf{x}_{f})p(\textbf{x},t) + \Omega _{q\rightarrow p} \delta (\textbf{x} - \textbf{x}_0)q(\textbf{x},t)\nonumber \\ \frac{\partial q}{\partial t}&= - \nu (\nabla \cdot (\textbf{v}q)) + \Omega _{p\rightarrow q}\delta (\textbf{x} - \textbf{x}_{f})p(\textbf{x},t) - \Omega _{q\rightarrow p} \delta (\textbf{x} - \textbf{x}_0)q(\textbf{x},t)\\ \frac{\partial u}{\partial t}&= D\Delta u - \gamma u + A q(\textbf{x},t) e^{-||\textbf{x}-\textbf{x}_f||^2}\nonumber \end{aligned}$$

Here, $\alpha$ is the diffusion coefficient for the foraging ants and $\sigma$ describes the sensitivity of the foraging ants to the pheromone. Mathematically, $u(\textbf{x},t)$ can be viewed as a potential. Collectively, these two terms describe the continuum analog of the unbiased random walk of foragers in the absence of pheromone and the biased random walk of foragers in the presence of a pheromone field. The transition parameters $\Omega _{p \rightarrow q}$ and $\Omega _{q\rightarrow p}$ are the constants governing the conversion of foraging ants to carrying ants and vice versa, respectively. The multiplying Dirac delta functions impose that the transitions can only occur at a food source or the nest. The coefficient $\nu$ describes the velocity of a carrying ant, and $\textbf{v} \equiv \frac{\textbf{x}_0 - \textbf{x}}{||\textbf{x}_0 - \textbf{x}||}$ is a unit vector directed at the nest. This constant velocity advection term describes the returner ants’ beeline return to their nest from a food source. The dynamics for $u(\textbf{x},t)$ are continuum analogs of the dynamics for $c(\textbf{x},t)$ shown in Eq. (1). We prescribe homogeneous Neumann conditions for $p(\textbf{x},t)$ and $q(\textbf{x},t)$ so that, like the lattice simulation, ants are conserved. For $u(\textbf{x},t)$ we prescribe homogeneous Fourier-type conditions. For initial data, we prescribe $p(\textbf{x},t) = \delta (\textbf{x}-\textbf{x}_0)$ and zero for $q(\textbf{x},t)$ and $u(\textbf{x},t)$.

The solutions to Eq. (2) will describe the probability densities for a single foraging ant ($p(\textbf{x},t))$ and a single carrying ant ($q(\textbf{x},t)$) and will also describe the densities of foraging and carrying ants in the macroscopic limit (i.e., the large number of ants limit).

**Fig. 5:**
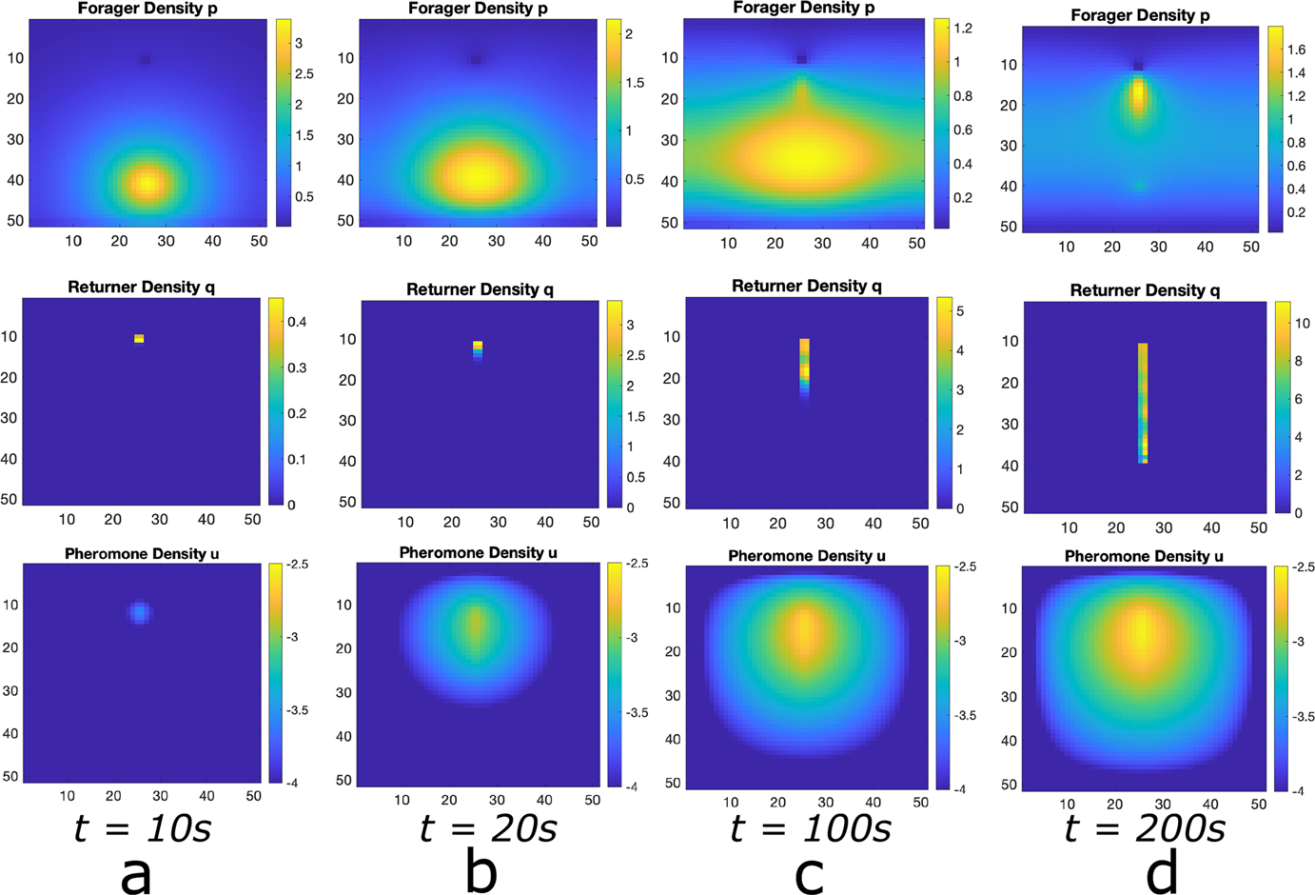
Numerical Simulation of Macroscopic PDE System. Simulated Eq. (2) for forager density *p*, returner density, *q*, and pheromone density, *u*, (plotted in log scale)using the Alternating Direction Implicit Method (ADI). **a** Initial configuration with a bump function of foragers and essentially no returners or pheromone, **b** food source is discovered and pheromone is released as returners move toward the nest. **c** the trail of foragers and returner develops at intermediate times. **d** the trail is well developed. This shows that the perturbation from the homogeneous solution discovered analytically above actually leads to the trail formation as at least a quasi-stable state

**Fig. 6:**
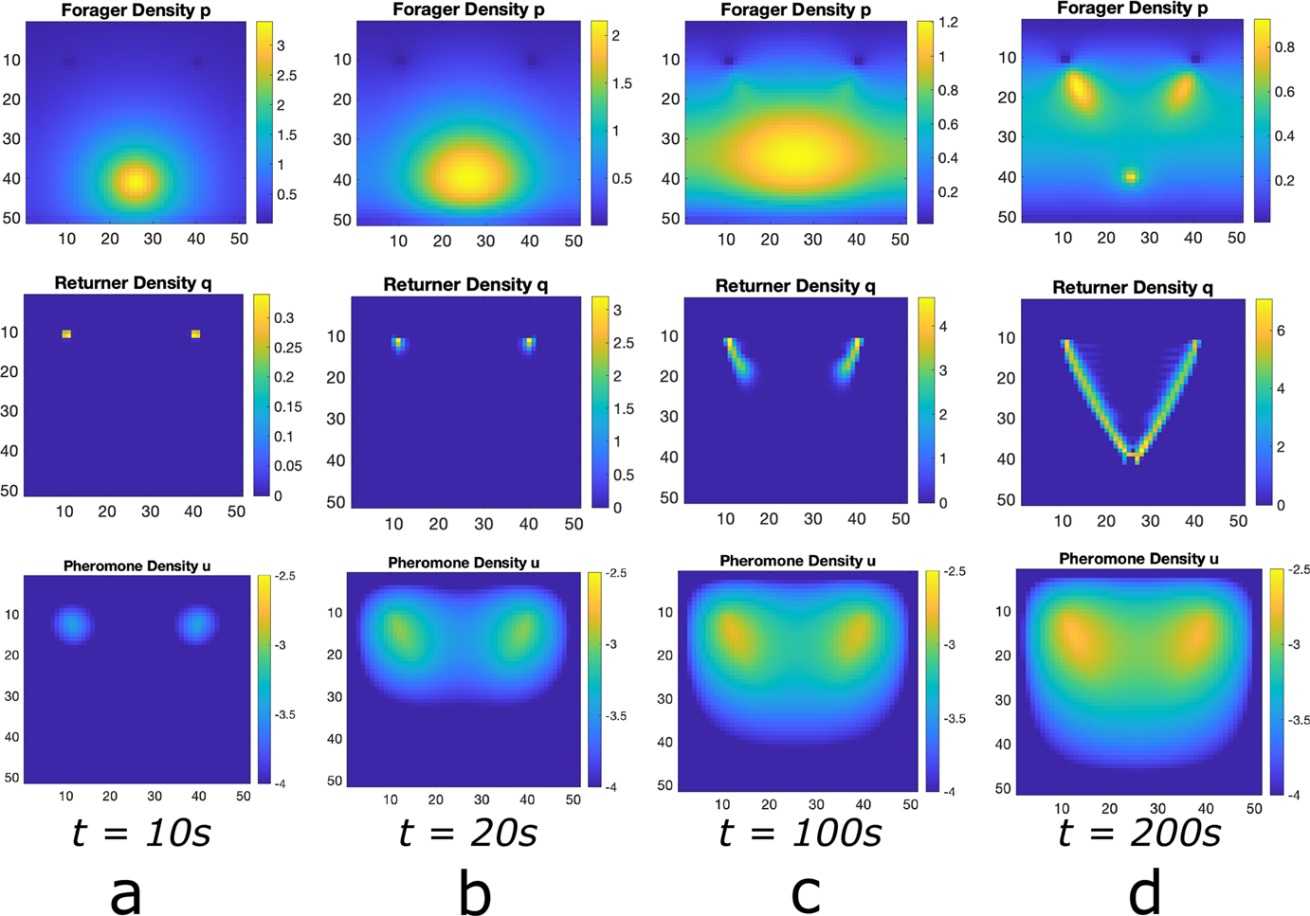
Numerical Simulation of Macroscopic PDE System for Multiple Food Sources. Simulated Eqn. (2) for forager density *p*, returner density, *q*, and pheromone density, *u*, (plotted in log scale) using the Alternating Direction Implicit Method (ADI). **a** Initial configuration with a bump function of foragers and essentially no returners or pheromone, **b** food source is discovered and pheromone is released as returners move toward the nest. **c** the trail of foragers an returner develops at intermediate times. **d** the trail is well developed. This shows that the master equation is capable of handling multiple food sources

**Fig. 7:**
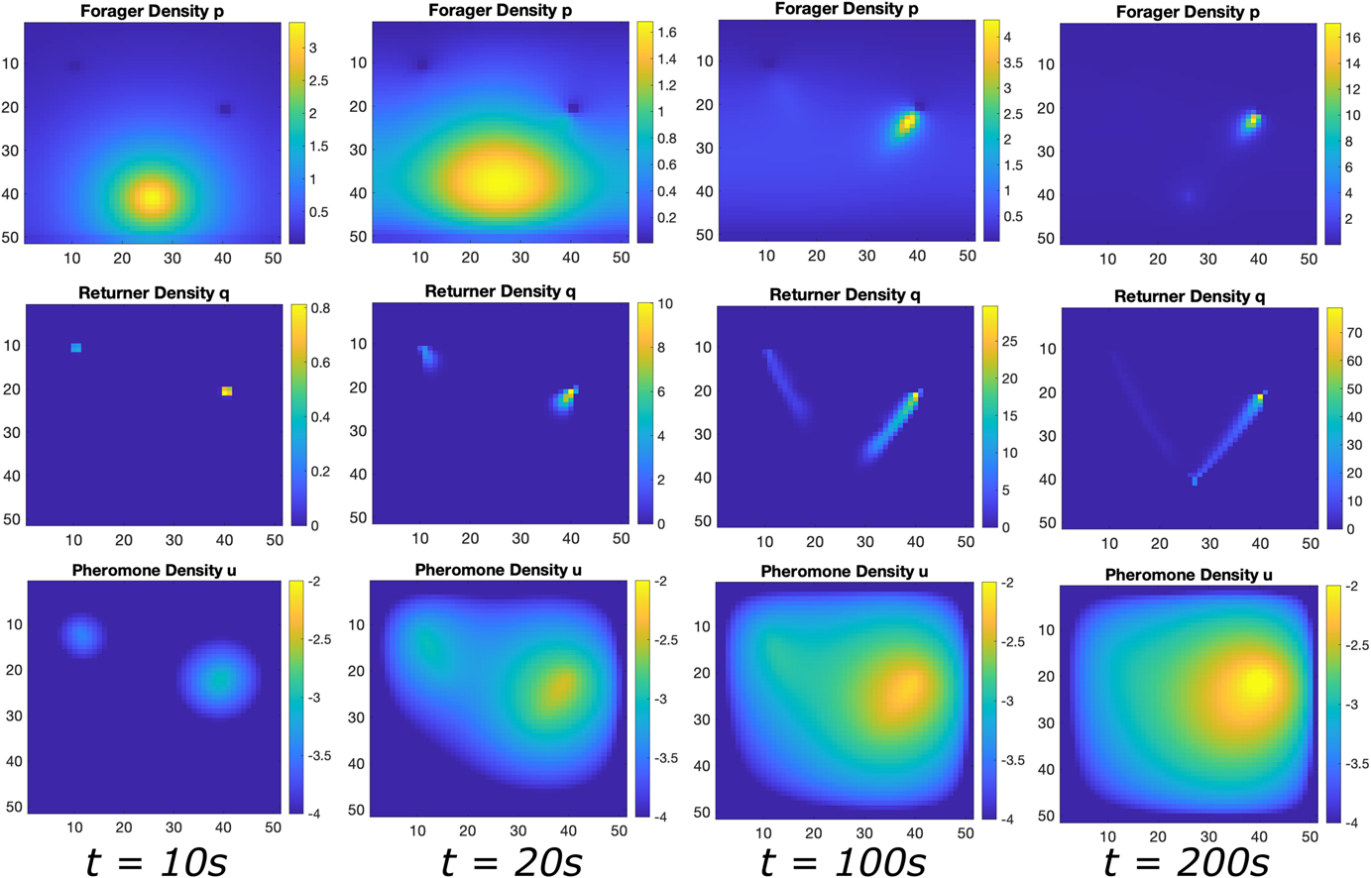
Numerical Simulation of Macroscopic PDE System for Multiple Food Sources at Unequal Distances. Simulated Eqn. (2) for forager density *p*, returner density, *q*, and pheromone density, *u*, (plotted in log scale) using the Alternating Direction Implicit Method (ADI). **a** Initial configuration with a bump function of foragers and essentially no returners or pheromone, **b** first food source is discovered and pheromone is released as returners move toward the nest. **c** the trail of foragers an returner develops at intermediate times to both food sources. **d** the trail is well developed at the closer food source and the trail to the farther food source evaporates naturally due to no ant visits

### Macroscopic PDE model results

Here we first establish that the results of the macroscopic model coincide with the lattice model results before performing a linear stability analysis showing that the presence of food sources is the key factor in establishing ant trails.

**Comparison with Lattice Model.** In Figs. 5, 6 and 7, we show solutions of Eq. (2) for three distinct situations corresponding to the results shown in Figs. 2, 3 and 4. The macroscopic PDE model solutions are qualitatively similar to the numerical solution to the lattice model simulations in all cases. In Fig. 5, we show foraging ant dynamics in an environment with a single food source. Here, following a transient Gaussian-distribution phase (Fig. 5a–c), all ants converge to a single trail connecting ant nest to the food source. In Fig. 6, we show foraging ant dynamics in an environment with two food sources equidistant from the ant nest. We see here that the stationary state consists of two trails that form connecting the nest to the food sources. Finally, in Fig. 7, we show foraging ant dynamics in an environment with two food sources, each located a different distance away from the nest. As in the lattice simulations (see Fig. 4), following a transient quasistationary phase where two trails form, the stationary state consists of a single trail connecting the nest to the proximal food source.

**Linear Stability Analysis.** Here we use a combination of analytical and numerical investigation to provide evidence that the presence of a food source in the domain of interest may promote the formation of ant trails. This is true in the sense that the homogeneous solution will be shown to be unstable for a range of key parameter values. Importantly, our results will correspond to an averaged PDE system (see Eq. (4) below). We do not assert that the stationary states of this system correspond to that which should be observed in the stochastic lattice model. Rather, we analyze Eq. (4) as a means to inform the full macrosopic PDE and the stochastic lattice model of parameters that can promote trail formation.

In the absence of food, Eqs. (2) reduce to

3 $$\begin{aligned} \frac{\partial p}{\partial t}&= \alpha \Delta p \nonumber \\ \frac{\partial q}{\partial t}&= 0\\ \frac{\partial u}{\partial t}&= 0 \nonumber . \end{aligned}$$

In the absence of a food source, foraging ants never become carrying ants and thus never produce pheromone. Thus, carrying ant dynamics and pheromone dynamics are trivial. Initial data then yield the homogeneous equilibrium $p_{h,0} (\textbf{x}) = \frac{1}{MN}$, $q_{h,0}(\textbf{x}) = 0$, and $u_{h,0}(\textbf{x}) = 0$.

We next show that the presence of food promotes a nonhomogeneous equilibrium to emerge. We equate this emergence with the formation of an ant trail from the nest to the food source. To do this, we average the coefficients in Eq. (2) over all of space so that coefficients are space-independent. This yields the equations

4 $$\begin{aligned} \frac{\partial p}{\partial t}&= \alpha \Delta p - \sigma \nabla \cdot ((\nabla _{\textbf{x}}u)p) - \frac{\Omega _{p\rightarrow q}}{MN} p + \frac{\Omega _{q\rightarrow p}}{MN} q \nonumber \\ \frac{\partial q}{\partial t}&= -\nu \left( \nabla \cdot \left( \frac{\textbf{x}_0-\textbf{x}_f}{||\textbf{x}_0-\textbf{x}_f||} q\right) \right) + \frac{\Omega _{p\rightarrow q}}{MN} p - \frac{\Omega _{q\rightarrow p}}{MN} q \\ \frac{\partial u}{\partial t}&= D \Delta u - \gamma u + \frac{A\pi }{4MN} E(\textbf{x}_f)q, \nonumber \end{aligned}$$

where we take $\textbf{x}_f = \langle x_{f1}, x_{f2}\rangle$ and $E(\textbf{x}_f) \equiv (\text {erf}(x_{f1}) - \text {erf}(x_{f1}-N))(\text {erf}(x_{f2}) - \text {erf}(x_{f2}-M))$. The homogeneous and nonhomogeneous stationary states of Eq. (4) will not correspond exactly to the stationary behavior of Eq. (2) or of the stochastic lattice model. However, we will use the structural similarity of Eqs. (4)–(2) divulge the impact various parameters impart on the destabilization of a homogeneous equilibrium of Eq. (4). We will use this information to inform the role various related parameters play in the destabilization of homogeneous stationary states in Eq. (2) and the stochastic lattice model. We identify the destabilization of the homogeneous state in Eq. (4) as the onset of trail formation.

A homogeneous equilibrium for Eq. (4) is given by

$$\begin{aligned} p_h(\textbf{x}) = \Gamma \frac{MN}{\Omega _{p\rightarrow q}}, \quad q_h(\textbf{x}) = \Gamma \frac{MN}{\Omega _{q\rightarrow p}},\quad u_h(\textbf{x}) = \Gamma \frac{A\pi }{4\gamma \Omega _{q \rightarrow p}}E(\textbf{x}_f), \end{aligned}$$

where $\Gamma \equiv \frac{1}{(MN)^2} \left( \frac{1}{\Omega _{p\rightarrow q}} + \frac{1}{\Omega _{q \rightarrow p}}\right) ^{-1}$. Observe that by taking the limits $\Omega _{p\rightarrow q} \rightarrow 0$, $\Omega _{q \rightarrow p} \rightarrow \infty$, and $E(\textbf{x}_f) \rightarrow 0$, we obtain the homogeneous equilibrium of the system without food: $p_{h,0} = \frac{1}{MN}$, $q_{h,0} = 0$, $u_{h,0} = 0$. The equations are thus self-consistent.

Following a common approach, we will linearize Eq. (4) about the homogeneous equilibrium and study the spectrum of the resulting linear operator. We consider the case where *M*, *N* are large relative to the distance between the nest and food source so that boundary effects are negligible for trail formation dynamics. This is a reasonable assumption because the trail formation in the mechanism studied in this paper is fueled through diffusive coupling. Large distances render diffusive coupling inefficient. Hence, we consider Eq. (4) in the unbounded domain $\mathbb {R}^2$.

We assume a solution of the form

$$\begin{aligned} p(\textbf{x},t)&= p_h(\textbf{x}) + \varepsilon p_1(\textbf{x},t)\\ q(\textbf{x},t)&= q_h(\textbf{x}) + \varepsilon q_1(\textbf{x},t)\\ u(\textbf{x},t)&= u_h(\textbf{x}) + \varepsilon u_1(\textbf{x},t), \end{aligned}$$

where $0 < \varepsilon \ll 1$ is a small parameter quantitating the perturbation imparted on the homogeneous solution. We look for plane wave solutions with wavevector $\textbf{k} \equiv \langle k_1, k_2 \rangle$: $p_1(\textbf{x},t) = \tilde{p}(\textbf{k})\text {exp}\left( i\textbf{k}\cdot \textbf{x} + \lambda t\right)$, $q_1(\textbf{x},t) = \tilde{q}(\textbf{k})\text {exp}\left( i\textbf{k}\cdot \textbf{x} + \lambda t\right)$, and $u_1(\textbf{x},t) = \tilde{u}(\textbf{k})\text {exp}\left( i\textbf{k}\cdot \textbf{x} + \lambda t\right)$. Substituting into Eq. (4) and collecting $O(\varepsilon )$ terms yields the linear system $\textbf{A}|p\rangle = |0\rangle$, where

$$\begin{aligned} \textbf{A}\equiv \left( \begin{array}{ccc} \lambda + \alpha ||\textbf{k}||^2 + \frac{\Omega _{p\rightarrow q}}{MN} & -\frac{\Omega _{q\rightarrow p}}{MN} & -\frac{\Gamma \sigma MN}{\Omega _{p\rightarrow q}}||\textbf{k}||^2 \\ -\frac{\Omega _{p\rightarrow q}}{MN} & \lambda - i\nu (\frac{\textbf{y}-\textbf{x}_f}{||\textbf{y}-\textbf{x}_f||} \cdot \textbf{k}) + \frac{\Omega _{q\rightarrow p}}{MN} & 0 \\ 0 & -\frac{A\pi }{4MN}E(\textbf{x}_f) & \lambda + \gamma + D ||\textbf{k}||^2 \end{array}\right) \end{aligned}$$

and $|p\rangle \equiv (\tilde{p}(\textbf{k}),\tilde{q}(\textbf{k}),\tilde{u}(\textbf{k})^T$. To ensure $|p\rangle$ is nontrivial, we set $\text {det}(\textbf{A}) = 0$. This yields an eigenvalue problem yielding a dispersion relation between $\lambda$ and $\textbf{k}$. The goal is to determine conditions under which $\text {Re}[\lambda (\textbf{k})] > 0$, meaning the homogeneous solution is unstable under a perturbation with the specified wave vector.

**Fig. 8:**
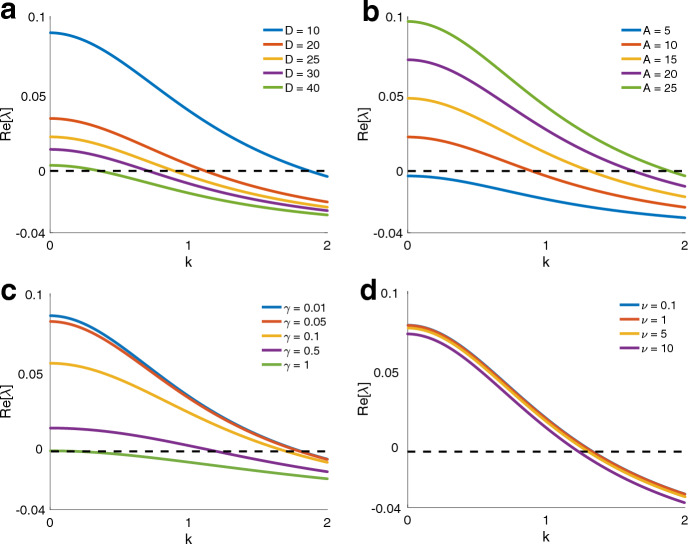
How $\lambda$ varies with wavevector $\textbf{k}$. This is a projection of the dispersion relation onto one of the components of $\textbf{k}$. The dispersion relation is symmetric with respect to the wavevector components. **a** Increasing the diffusion rate of the pheromone signal stabilizes the homogeneous steady state. **b** Increasing the quantity of pheromone secreted per unit time destabilizes the homogeneous steady state. **c** Increasing the degradation rate of the pheromone has the same effect as decreasing the pheromone secretion rate. **d** Varying the speed of returning ant motion marginally affects stability of the homogeneous equilibrium

**Fig. 9:**
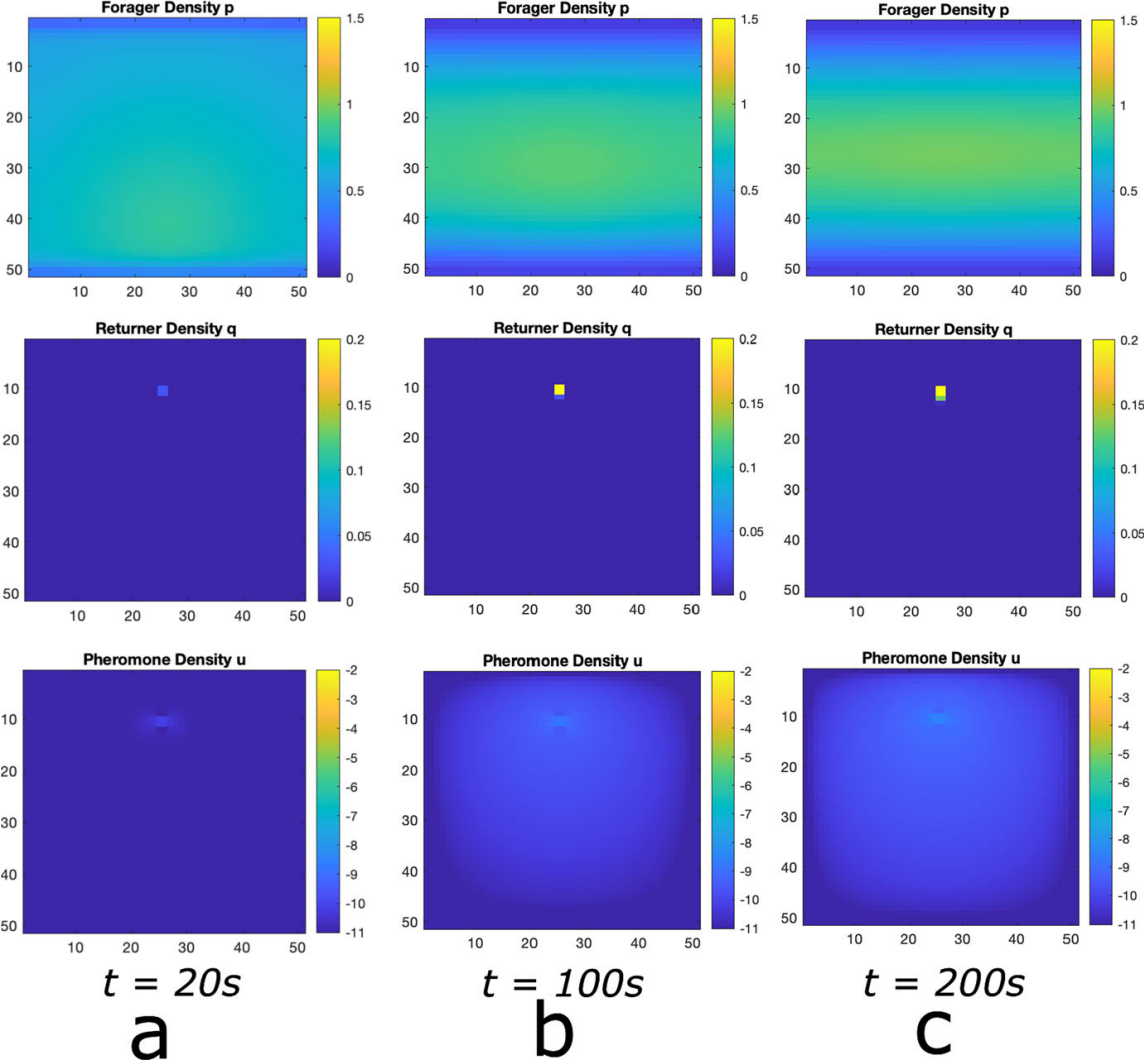
Simulations for macroscopic Eq. (2) showing stable homogeneous state. Independent of time the foraging and returning densities are near uniform. We note that one point where the returning density spikes is the food source where every time step foragers near there are still converted to returners. This also leads to pheromone being slightly higher around the food source, but overall quite close to a uniform distribution

Although in theory $\lambda (\textbf{k})$ is computable, the expression is long and unwieldy. We use MATLAB to compute the dispersion relation. The dispersion relation is symmetric with respect to $k_1$, $k_2$. Thus, in Fig. 8, we show the projection of the dispersion surface onto $\lambda k_1$–space. Under the prescribed parameters, clearly $\lambda (\textbf{k}) > 0$ for a range of wavevectors, indicating instability of the homogeneous solution to low frequency perturbations. The homogeneous solution is stable under high frequency perturbations. However, the instability to low frequency perturbations is what we interpret as the onset of ant trails (nonhomogeneous solution).

To investigate this further, we show in Fig. 8a how the dispersion relation changes when *D*, the diffusion coefficient of the pheromone signal, is altered. We find that as *D* is increased, the homogeneous steady state is stabilized. This is easy to understand: when *D* is large, diffusion dominates pheromone dynamics and diminishes any observable gradient in the pheromone concentration field. With a uniform pheromone distribution, ants will be unable to form trails.

In Fig. 8b, we show how the dispersion relation changes as *A*, the quantity of pheromone secreted by an ant per unit time, is altered. We find that as *A* is increased, the homogeneous steady state is destabilized. If not enough pheromone is secreted, then clearly there will not be enough pheromone for ants to sense and modify their motion. Indeed, for $A = 5$, we observe an unconditionally stable stationary state. Trails form only when enough pheromone is secreted into the environment.

In Fig. 8c, we show how varying $\gamma$, the pheromone degradation/evaporation rate, affects the dispersion curves. Increasing $\gamma$ stabilizes the homogeneous steady state. This is intuitive—increasing $\gamma$ is in effect equivalent to decreasing *A*. In Fig. 8d, we vary $\nu$, the velocity of returning ants, and observe how that affects the dispersion curves. We observe effectively no dependence on the velocity of returning ants. This parameter effectively alters the timescale on which the stationary states are reached but does not alter stability properties.

In Fig. 9 we briefly present the results of simulations for equation Eq. (2) using a selection of parameter values where the linear stability analysis predicts the homogeneous state will be stable in the presence of food (e.g., large diffusion rate of the pheromone *D* and low pheromone deposition amount by the returning ants *A*).

Thus, our macroscopic PDE model results coincide with the stochastic lattice model results. Moreover, the PDE model is amenable to analysis, and it makes explicit the crucial role the presence of food in an environment plays on the onset of emergent spatiotemporal ordering in ants. Linear stability analysis allows us to investigate the impact of various parameters upon the stability of the equilibrium with trails connecting the ant nest to food sources.

## Discussion

We have introduced a novel lattice model describing foraging ant dynamics that captures only basic essential features of forager ant dynamics and forgoes more detailed interactions. Indeed, the driving mechanism we incorporate in our model is chemotaxis and is not specific to ants. But we have shown that it is sufficient to describe complex emergent spatiotemporal ordering in foraging ants. Importantly, the emergent ordering is from the perspective of the foraging ants and not the returning ants. While returning ants tend to “remember” the location of the nest and make a beeline towards it Wehner (2003); Wehner et al. (2016), the foragers begin to self-organize upon coming into contact with pheromone secreted by the returning ants. Thus, the inclusion of returning ants in trails connecting the ant nest to a food source is encoded into the model, but the convergence of foraging ants to those same trails is an emergent property.

Our model reproduces results produced by more detailed agent-based models, and even extends some results. Specifically, our model admits multiple trails from the ant nest to multiple distinct food sources, improving on previous models which could not sustain multiple ant trails in the presence of multiple food sources (e.g., Ryan (2016)).

While multiple food sources can be visited simultaneously in the agent-based model, after initial discover the colony tends to favor one source over another for efficiency. This is often the closer food source because it is visited more frequently and therefore has a stronger pheromone gradient. In contrast, the lattice model introduces the biased random walk where ants still have the ability to visit any previous sites encountered with probability proportional to the local pheromone concentration gradient. Often times the failure to obtain multiple trails over a significant period of time can be attributed to the presence of both gradients in the equations of motion resulting in a relative weighting of contributions leading to an averaged effective trajectory for each ant. That is, an ant in the agent-based model may weight contributions of the gradients in such a way as to follow a trail to a location between food sources that does not contain any food. In the lattice model the foraging ants leaving the nest choose a direction based on a weighted probability and that gets reinforced as they move along a particular trail.

Interestingly, our model shows strong prejudice when asymmetry is present in the system. Namely, when multiple food sources are present, but are not equidistant from the nest, at equilibrium all ants are in the trail connecting the nest to the nearest food source. The implication is that sending ants to extract food from farther food sources is inefficient. This maps to experimental observations describing the efficiency of ants (Wehner 2003; Wehner et al. 2016). Initially, the ants form trails to all the available food sources. This stems from the fact that in the absence of a pheromone concentration field, foragers perform a 2D random walk. The multiple food trails manifest as a quasi-stationary state. Once pheromone from the nearest food source becomes perceptible, all ants converge to the trail forming between the nest and the nearest food source. When food sources are equidistant, ant trails to each food source manifest in the equilibrium structure.

The simplicity of our model allows for analysis. We describe a macroscopic PDE model that captures essential features of stochastic lattice model to illuminate model properties. This PDE makes explicit the crucial role the presence of food sources plays in the onset of ant trails. Furthermore, linear stability analysis of the PDE model facilitated a computational study delineating how diffusion rate of the secreted pheromone, pheromone secretion rate, pheromone degradation rate, and returning ant velocity can destabilize homogeneous equilibria and make trail formation possible. In particular, our computation of dispersion curves demonstrates that the presence of food is a *necessary* but not *sufficient* condition for the onset of trails. Thus, the amenability of our lattice model allows us to unveil key biophysical parameters that govern ordering in foraging ant dynamics.

The directness of our model facilitates a number of future extensions we hope to explore. First, we hope to explore the impact of terrain upon trail formation. To incorporate this, we can prescribe space-dependent hopping rates in our lattice model—smaller hopping rates capture more difficult terrain and larger hopping rates represent flat terrain. Second, we hope to explore the impact of competition between distinct ant colonies on trail formation. Third, we hope to investigate the impact of environmental factors (like rain, which can disrupt pheromone gradient formation) and predators upon trail formation. In the latter case, groups of ants are more prone to predator attacks than isolated ants, causing a cost-benefit problem between ants grouping together to acquire food and isolating away from one another to minimize death due to predation.

Other recent work found that ants actively avoid crowded food sources to facilitate trail formation to other food sources (Wendt et al. 2020). The driving mechanism there was a combination of chemical interactions and individual interactions. We can incorporate individual interactions by modeling the stochastic lattice model as a modification of a TASEP (Bressloff and Karamched 2016; Täuber 2014; Derrida et al. 1993; Chou et al. 2011) or by including a Lennard–Jones potential (Fischer and Wendland 2023; Ryan 2016; Gardner and Radin 1979) into the macroscopic PDE formulation.

Finally, we note that an alternative modeling paradigm has been explored by others to study pheromone detection and trail formation by ants: velocity-jump models (Amorim et al. 2019; Malíčková et al. 2015; Ramirez et al. 2018). In such models, the response of an ant to a greater pheromone concentration is to *turn toward* the direction of greater pheromone concentration. In particular, orientations of ants are tracked in time and altered in response to pheromone. This is in contrast to our modeling paradigm, where an ant *moves towards* the direction of higher pheromone concentration (position-jump process). Indeed, such models result in more robust, biological realistically results than position-jump models (Amorim et al. 2019; Malíčková et al. 2015; Ramirez et al. 2018; Mokhtari et al. 2022; Vela-Pérez et al. 2015; Fontelos and Friedman 2015). For example, in Amorim et al. (2019) a velocity-jump model was used to show that the positioning of ant antennae at the front of their bodies is necessary for ants to have the ability to follow a pheromone signal. Furthermore, velocity-jump models have the advantage of not needing a precise definition of $\mathcal {W}_a$ as we have constructed to avoid ants becoming stuck at a local maxima of pheromone. This is because in velocity-jump models ants are able to follow pheromone trails with a well-defined crest but no clear lengthwise gradient.

Moreover, such models have been shown to coincide with Weber’s Law (Smeets and Brenner 2008; Cammaerts and Cammaerts 2020). Weber’s law as it pertains to ants roughly states that if *L* is a pheromone concentration on the left of an ant and *R* is the analog on the right, the individual response is proportional to $(L-R)(L+R)^{-1}$ (Amorim et al. 2019). Weber’s law as applied to ant movement was shown to hold experimentally in a convincing work (Perna et al. 2012). Varying ant orientation in response to pheromone (velocity-jump models) is a direct representation of Weber’s Law.

Our model does not explicitly account for Weber’s Law in ant motion. Indeed, ants in our stochastic lattice model do not have orientation. However, the net effect of Weber’s Law is still captured in that the motion of ants in response to pheromone gradients are preferentially towards higher concentrations with a nonzero probability of motion in other directions. This results in “messy” trail formation, wherein there is a definitive trail with a sporadic distribution of individual ants about the trail. This was shown in Malíčková et al. (2015), and our model results behave similarly.

Indeed, the results presented in Malíčková et al. (2015) are similar in flavor to our work in this manuscript. There, the authors use a velocity-jump model to understand trail formation between an ant colony’s nest and a food source. They further assume that ants secrete two orthogonal pheromones: when they are foraging they secrete one and follow the other whereas when they return home they do the opposite. Multiple food sources are also explored, and, as in our model, persistent trails are formed connecting the nest to all available food sources provided they are equidistant from the nest. The case of food sources of varying distances from the nest is not explored. Instead, they determined that the diffusion rates of the two pheromones affect the time required to find a food source versus the nest—a nest pheromone with a large diffusion coefficient renders the nest more difficult to find than if the diffusion coefficient were smaller. The modeling assumptions there are fundamentally different from our model’s, so a direct comparison of results is not possible. However, qualitatively, the two models behave similarly.

What is missing in these velocity-jump models—and what our modeling framework facilitates—is the description of the macroscopic structure of the system in the form of a PDE. Although the PDE we use to compare results with the stochastic lattice model is not systematically derived from the stochastic lattice model, it captures essential features of it and its solutions qualitatively match the stochastic lattice model. Analysis of the PDE allowed us to determine the impact of various biophysical parameters on stability of the homogeneous state. Hence, our model is amenable to analysis.

This naturally leads us to have another avenue for research in the future. It would be interesting to derive an effective PDE that describes the macroscopic structure of the velocity-jump process as it pertains to ants. In principle, it is possible to construct a diffusion limit of such a velocity-jump process consisting of an anisotropic diffusion tensor describing ant dynamics Othmer and Hillen (2000); Newby and Keener (2011). We hypothesize that the structure of such an effective equation will be similar structurally to Eq. (2). We hope to explore this further in future work.

## Footnotes

1 One can find sample versions of the codes used to produce the results of this manuscript in Ryan and Karamched (2024).

2 In principle, such a model could be implemented on an unbounded domain and the results of this manuscript will still hold due to 2D random walks being recurrent. Thus, there would be no loss of ants. For the sake of computational ease, we formulate the model on a finite lattice.

3 For a diffusion-degradation process with diffusion coefficient *D* and decay rate $\gamma$, the diffusion correlation length is $\sqrt{D/\gamma }$ (Bressloff et al. 2017; Karamched and Bressloff 2017).
